# Effect of encapsulation on electronic transport properties of nanoscale Cu(111) films

**DOI:** 10.1038/s41598-019-40193-6

**Published:** 2019-03-05

**Authors:** Prashant P. Shinde, Shashishekar P. Adiga, Shanthi Pandian, K. Subramanya Mayya, Hyeon-Jin Shin, Seongjun Park

**Affiliations:** 1 Materials Simulation (SAIT-India), Samsung R&D Institute, Bangalore, India; 2 Inorganic Material Lab, Samsung Advanced Institute of Technology, Suwon, 433-803 Republic of Korea

## Abstract

The stiff compromise between reliability and conductivity of copper interconnects used in sub-nanometer nodes has brought into focus the choice of encapsulation material. While reliability was the primary driver so far, herein, we investigate how electronic conductivity of Cu(111) thin films is influenced by the encapsulation material using density functional theory and Boltzmann transport equation. Atomically thin 2D materials, namely conducting graphene and insulating graphane both retain the conductivity of Cu films whereas partially hydrogenated graphene (HGr) results in reduction of surface density of states and a reduction in Cu film conductivity. Among transition metal elements, we find that atoms in Co encapsulation layer, which essentially act as magnetic impurities, serve as electron scattering centres resulting in a decrease in conductivity by at least 15% for 11 *nm* thick Cu film. On the other hand, Mo, Ta, and Ru have more favorable effect on conductivity when compared to Co. The cause of decrease in conductivity for Co and HGr is discussed by investigating the electronic band structure and density of states. Our DFT calculations suggest that pristine graphene sheet is a good encapsulation material for advanced Cu interconnects both from chemical protection and conductivity point of view.

## Introduction

**Figure 1:**
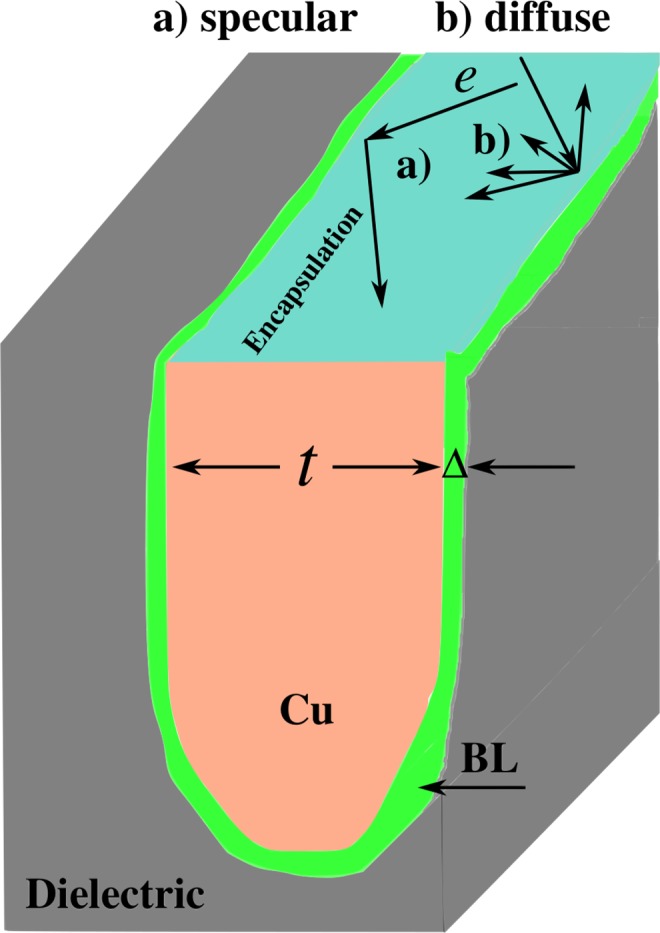
Schematic of encapsulated Cu interconnects in SiO_2_ dielectric. The thickness of conventional barrier layer, BL, (Δ) affects the overall thickness (*t*) of the Cu interconnect. The formed interface between the conducting materials leads to either specular (**a**) or diffuse (**b**) scattering of electrons, *e*.

The continued downscaling of integrated circuits to increase the density of transistors on a chip has necessitated reduction in lateral dimensions of copper (Cu) interconnects to values well below the mean free path for electrons (40 *nm* in Cu). This has posed serious challenges to further downscaling because of increased electrical resistivity and inter-metal capacitance that result in cross-talk and dynamic delay issues. Additionally, severe reliability concerns arise due to Joule heating that accentuates the problems of electromigration and Cu diffusion^1,2^ into the dielectric material. The increase in electrical resistivity can be controlled by growing uniform large grains of Cu. The smooth surface of large grains promotes specular scattering. In state-of-the-art microelectronics circuits, Cu interconnects are always used with a thin interlayer film between itself and the interlayer dielectric that serves as a Cu diffusion barrier, an adhesion promoter, and to arrest electromigration driven void formation and eventual loss of contact. In addition to the barrier layer, an encapsulation layer on the planarised surface is also used to electrically and chemically isolate the Cu lines (Fig. 1). Traditionally, SiCN-like materials with relatively low dielectric constants are used to achieve the purpose^3,4^.

In this regard, several candidate materials have been considered for Cu encapsulation. Among them, cobalt (Co) and Co alloys have emerged as promising candidates for complete encapsulation of advanced Cu interconnects^5^. Indeed, recent studies^6^ have shown that smooth and highly conformal Co films can improve the reliability and thermal stability of Cu interconnects. Alternative to Co encapsulation, interesting research has also been conducted with graphene as an encapsulation material, where graphene is shown to be effective in reducing electromigration^7,8^. While the choice of encapsulation material is primarily driven by reliability improvement and compatibility of the process with the 3D integration technology, an overlooked opportunity is to improve Cu conductivity through optimal encapsulation layer. The stiff trade-off in interconnect figures of merit at small scales mandates solutions that simultaneously addresses multiple challenges. Given the limitations on how much current density nanoscale cross-sections can support, one can think of the encapsulation layer that also positively affects the electronic structure of surface copper atoms and improves effective conductivity. Recently, for example, it was reported^9^ that the conductance of Cu could be enhanced by graphene capping and it was argued that graphene can reduce inelastic scattering at interface between Cu and graphene and thereby increasing conductivity. However, the experimental work was performed on thick Cu film of around 200 *nm* that are less affected by surface inelastic scattering than nanoscale interconnects^10^. Thus, it is not clear if graphene encapsulation enhances or preserves Cu conductivity at the nanoscale.

In fact, a more fundamental question to ask is what materials or material properties are desirable for encapsulation purposes purely from the point of view of Cu conductivity? How does graphene, a conducting 2D material compare with Co? In addition to Co and graphene there has been a lot of interest in other platinum-group metals. Especially, Ru has emerged as a promising candidate as it shows a very low solubility in copper and strong adhesion to Cu^11,12^. As we optimise interfaces for reliability and conductivity, it is important to understand how encapsulation materials affect the transport properties. It is well established, for example, that the conductivity of Cu interconnects is sensitive to the electronic properties of barrier material^13–16^. Especially, the density of states (DOS) matching between Cu film and the barrier material and behavior of conduction electrons at the interface. Are there general rules with respect to the nature of chemical bonds formed between Cu and the encapsulating material or transport property of the encapsulating material and how they affect the conductivity of Cu films? There has not been systematic investigations on the effect of encapsulation on the electronic conductivity of Cu films.

Therefore, we investigate, within density functional theory (DFT) and semi-classical Boltzmann transport theory, the electronic transport properties of atomically smooth Cu(111) thin films upon encapsulation. We consider ideal thin films, with no line-edge roughness and no grain boundaries. The films are encapsulated with atomically thin layers of graphene (Gr), partially hydrogenated graphene (HGr), Graphane, cobalt (Co), ruthenium (Ru), tantalum (Ta), and molybdenum (Mo). These materials are being explored as promising reliable protective coatings as their atomically thin layers can be grown on various substrates. First, we investigate how the encapsulation materials affect the bonding nature and structural relaxation at Cu film surfaces. Then we probe the effect of different encapsulations by computing the transport coefficient, effective electronic conductivity (*σ*/*τ*), for Cu films with thickness ranging from 2 to 11 *nm*. We found that 2D materials, namely Gr and Graphane, retain the conductivity of bare Cu films whereas Co and HGr encapsulation reduce the conductivity. The effect of atomically thin layer of Ru encapsulation lies in between Co and Gr whereas an atomic layer of Mo or Ta shows favorable effects on conductivity compared to Co and Ru. The cause of modulation in conductivity for 2D materials and transition metal encapsulations is discussed by investigating the electronic band structure and DOS for a Cu film of thickness, *t* = 4 *nm*.

## Results and Discussions

### Film Encapsulation Schemes and the effect on film structure

**Figure 2:**
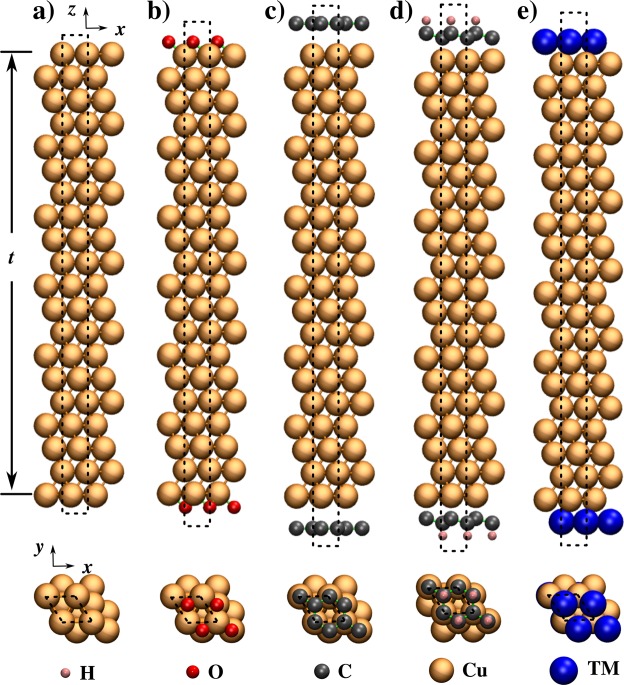
Side and top views of periodic boundary models of (**a**) bare Cu(111)-(1 × 1) thin film of thickness, *t* (**b**) oxygen coverage of 1 ML (**c**) graphene encapsulation (**d**) hydrogenated graphene encapsulation and (**e**) transition metal (TM = Co, Mo, Ta, and Ru) encapsulation. The simulation cell is indicated by dotted lines.

Atomically smooth surface promotes the developement of uniform highly textured large grains of Cu in the electroplated films. Smoother the surface of Cu grains, greater is the specularity. Therefore, in the present work we considered Cu films with atomically smooth surfaces. The models of ideal Cu thin film are constructed such that the atomic layers of the (111) crystal plane are perpendicular to the *z*-axis, and thus, there are two Cu (111) free surfaces. We considered (111) orientation because the formation energy for (111) surface is the lowest when compared to other crystallographic orientations and the (111) texture is often observed in electroplated Cu interconnects^17,18^. All atomic layers in the Cu film are optimized using DFT within VASP^19^. The atomic models of Cu(111)-(1 × 1) thin films are shown in Fig. 2, with the film thickness (*t*), in *z*-direction, varied from 2 to 11 *nm* while the corresponding number of atomic layers vary from 11 to 51. It is important to point out that the thickness of Cu films is larger than the Fermi wavelength of electrons in Cu (~0.50 *nm*)^20^ and is large enough to avoid any effects of interactions between the surfaces^21,22^. The periodicity in the *x*- and *y*-directions was fixed to the bulk DFT minimized value of 2.55 Å which corresponds to the distance between two Cu atoms in fcc bulk. This bond distance is in good agreement with the value 2.54 Å reported^23^ in a recent study. The calculated lattice constant for bulk Cu, 3.60 Å, is in excellent agreement with the experimental value of 3.61 Å at room-temperature^24,25^. In the bare Cu film (Fig. 2a), after geometry optimisation, the Cu-Cu distance changes by <3%. A tendency to reduce the bond distance to the neighbors is induced by morphological instability at the surface.

In addition to bare Cu films, we considered Cu films with surface oxide since the surface of nanosized Cu films is susceptible to oxidation and surface oxidation reduces film conductivity^26^. The Cu films get oxidised easily not only when it is in contact with air but also during its growth in a ultra-high vacuum chamber^27,28^. Figure 2b shows the presence of copper oxide (Cu-O) on the surfaces. Our simple slab model represents an initial oxidation stage with a low oxygen coverage on the surface. The oxygen coverage is ~1 ML. In the slab model with inversion symmetry, each O atom occupies the three-fold coordinated hcp site. The chemical bond between Cu and O is 1.85 Å and is in good agreement with the theoretical value reported by Cuong *et al*.^29^ and experimental value of 1.85 Å^30,31^. The formation of Cu-O is in general energetically stable^30^.

**Figure 3:**
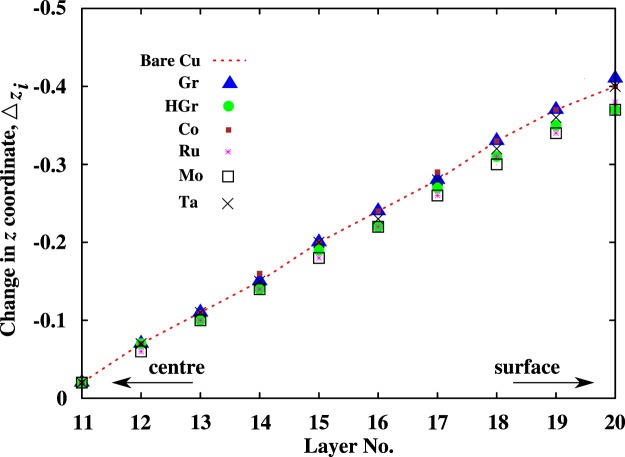
Calculated change in the *z* coordinate, ${{\rm{\Delta }}}_{{z}_{i}}$, of Cu atomic layers in 4 *nm* (20 atomic layers) thick film upon different encapsulations. The change in Å for the *i*^*th*^ layer is calculated w.r.t. its bulk position. Tabular data is provided in the Supplementary Information (Table S1).

Next we consider encapsulated Cu films. To model encapsulation, we placed a monolayer of an encapsulation material on the free surfaces. The encapsulation materials are divided into two groups. The first group contains 2D materials, namely Gr, HGr, and fully hydrogenated Gr called Graphane and the second group contains TMs viz. Co, Mo, Ta, and Ru. The Gr encapsulated Cu film as shown in Fig. 2c is modeled by fixing the lattice spacing of graphene to 2.55 Å. This corresponds to a strain in Gr lattice of 3.7%. A recent study by Giovannetti *et al*.^32^ showed that even with the lattice mismatch of ~4% the electronic properties of Gr remain intact. Due to weak van der Waals (vdW) interaction between a Cu film and Gr there is very little change in the atomic arrangement at the Cu surface when compared with bare Cu (see Fig. 3). The equilibrium distance between Gr and surface Cu atoms is close to 3.27 Å and matches well with the literature value of 3.26 Å^32,33^. The strong covalent bonding between carbon atoms in Gr offers a very high diffusion barrier against O penetration. A recent molecular dynamics simulations based study^29^ reported 20 eV energy barrier for O diffusion through graphene. Such a high energy barrier indicates that the diffusion is unlikely to happen. Atomic hydrogens when chemisorbed in an ordered configuration on graphene alters its geometrical and electronic structure^34,35^. In our DFT study with smallest unit cell, one H atom is adsorbed on a Gr sublattice leaving the other sublattice non-hydrogenated *i*.*e*. HGr. Geometry optimisation results in buckled graphene with C-C and C-H bond lengths of 1.54 and 1.09 Å, respectively. The longer C-C bonds are due to change in hybridisation from *sp*^2^ to *sp*^3^. The remaining non-hydrogenated C atom has an unpaired electron sitting in its *p*_*z*_ orbital. The equilibrium distance between HGr and Cu film is nearly 2.30 Å. Such a small distance could lead to a strong interaction between C[*p*_*z*_] and Cu[*s*] orbitals upon HGr encapsulation. In addition to graphene, the high electronic conductivity of Co (~1.61 × 10^7^ S/m^36^) has attracted a lot of attention as an encapsulating material for Cu interconnects. A recent study^6^ has demonstrated that Co encapsulation stabilises Cu against diffusion and electromigration. As Co is a magnetic material, the lattice constant of ferromagnetic hcp (fcc) Co is 3.3% (2.4%) smaller than that of bulk Cu. However, for an atomically thin Co layer on a Cu film, the comparison of the bulk phases is not necessarily relevant^37^. It is observed^38^ that for ultra-thin films of Co adsorbed on Cu surfaces, the fcc phase is most stable. Therefore, in our DFT simulations we assume pseudomorphic growth of Co and other transition metal (TM) films adapting the lateral spacing of the underlying substrate. One can expect small tensile strain at the Cu-Co interface. The under-coordinated Co atoms on the surface lead to a stronger in-plane bonding and thus shorter Co-Co bonds. For Co encapsulation modeled with spin polarisation, the calculated Cu-Co bond distance is 2.52 Å, which is shorter than Cu-Cu bond length in bulk copper but larger than Cu-Co bond distance, 2.49 Å, in spin unpolarised calculation. Similarly, upon Ru, Mo, and Ta encapsulation, the bond lengths of Cu-Ru, Cu-Mo, and Cu-Ta are 2.61, 2.71, and 2.80 Å, respectively. Such different bonding nature at Cu surface could affect the interface scattering resulting in changes in resistivity of Cu films.

Due to under-coordinated surface atoms, the bonding strength between the surface and subsurface atoms increases, resulting in an inward relaxation in the *z*-direction (${{\rm{\Delta }}}_{{z}_{i}}$). The effect of different encapsulation materials on structural relaxation of individual atomic layers in a 4 *nm* Cu(111) thin film w.r.t. the corresponding bulk positions is plotted in Fig. 3. Only changes on one side from the centre of the Cu film are indicated. The two surface layers correspond to layer number 1 and 20. A negative value indicates the overall change in the *z* coordinate of an atomic layer in the Cu film. We see that all encapsulation materials show similar inward relaxation. The overall change in the *z* coordinate is negligible in the centre of Cu film. This indicates that the bulk Cu atoms are less affected. The inward relaxation observed at the surfaces upon O adsorption is very similar to that in bare Cu film (not shown). Therefore, in the following section we investigate the effect of structural relaxation and the bonding nature of Cu with the TMs on the electronic and transport properties of the encapsulated Cu films.

### Electronic and Transport Properties

To calculate the transport properties of bare and encapsulated Cu films, first the optimised film structures were used to obtain electronic structures using VASP. The transport coefficient such as the electronic conductivity is calculated by solving the semi-classical Boltzmann transport equation as implemented in the Boltztrap code^39^. The code uses the band and *k*-dependent quasi-particle energies, as well as intra-band optical matrix elements and scattering rates, as input^40^. The relaxation time (*τ*) is dependent on both the band index and the *k* vector direction. Using Boltztrap, our computed effective electronic conductivity (*σ*/*τ*) value 1.72 × 10^21^ (Ω*ms*)^−1^ for bulk Cu at room temperature matches well with the theoretical value reported by Cuong *et al*.^29^. This gives the resistivity of bulk Cu to be *ρ* = 2.15 × 10^−8^ Ω*m* which agrees well with the experimental value of 1.72 × 10^−8^ Ω*m*^41^.

**Figure 4:**
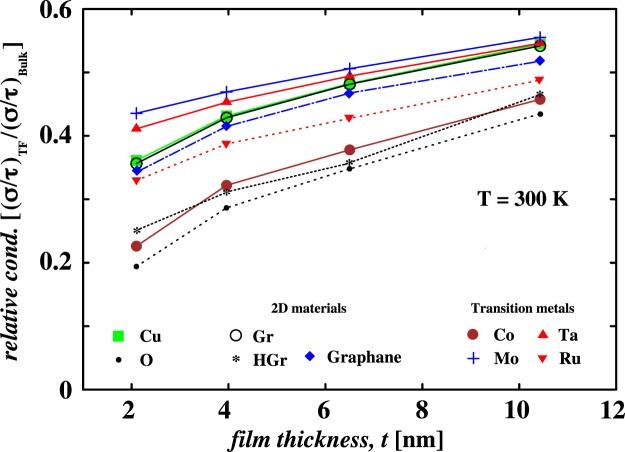
Relative electronic conductivity of Cu thin film (TF) as a function of film thickness *t* for different encapsulation materials. The effective electronic conductivity (*σ*/*τ*) calculated at room temperature is normalised by the bulk Cu conductivity value.

In Fig. 4, the relative conductivity of Cu films as a function of thickness is compared for different encapsulations. We mention that the relative conductivity, the ratio of effective electronic conductivity of a Cu film to the bulk effective electronic conductivity, is calculated with spin unpolarisation. An ultra-thin Cu film with thickness comparable to the Fermi wavelength of electrons in Cu (*λ*_*f*_ ~ 0.5 *nm*) can be viewed as a quantum well with infinite barrier height and width equal to film thickness. For such films the electron momentum is quantised and this results in properties dominated by quantum size effects. The Cu film with *t* = 2 *nm* shows the relative conductivity of ~0.36. With increase in film thickness to 4, 6, and 11 *nm* the relative conductivity increases to 0.43, 0.48 and 0.55, respectively (see green squares in Fig. 4). For bare Cu films, the relative electronic conductivity increases with film thickness due to reduced quantum effects. The smoother geometry at the surface results in a smooth surface electronic potential causing delocalisation of surface states parallel to the surfaces. Therefore, the surfaces of Cu films exhibit 2D free-electron-like states called Shockley surface states^42,43^. These surface states lie close to the Fermi energy (E_*f*_). Therefore, due to their significant contribution to DOS near E_*f*_^44^ and nearly free-electron-like nature, the Shockley surface states play a crucial role in the electronic transport. For better electronic transport through the Cu interconnects, the Shockley surface states must be preserved. However, in reality, undesirable oxidation of the surface influences the electronic structure of Cu films to a certain extent or even severely. Since the energies of the Cu *d*-orbitals and O *p*-orbitals lie very close, a strong hybridisation between Cu-O states leads to elimination of Cu surface states^45,46^. In Fig. 4 we show the effect of O adsorption on the calculated relative conductivity (see small filled circles). We see that O adsorption reduces the relative conductivity by ~45% for the thinnest film (*t* = 2 *nm*). The effect is less pronounced for thicker films. For the thicker film (*t* = 11 *nm*), the relative conductivity is reduced by 20% when compared with the bare Cu value. The diminishing effect of O adsorption can be understood in terms of overall effective width of Cu film. For thicker films, the effective width of Cu film would be larger. In contrast, for thinner film the effective width could be comparable to the Fermi wavelength of electrons in Cu leading to large modulations to the relative conductivity. With Gr encapsulation the electronic conductivity trend closely follows bare Cu. This is due to the weak vdW interaction between Gr and Cu film. Due to the weak interaction, the electronic structure of Cu film is well preserved and so the transport properties. Similarly, Graphane encapsulation preserves the electronic transport properties of the Cu films. The effect of Graphane encapsulation on the relative conductivity is indicated by blue diamonds. This is again an example of weakly bonded systems. Unlike Gr and Graphane encapsulation, the HGr encapsulation leads to reduction in the relative conductivity. For HGr encapsulated Cu film of thickness *t* = 2 *nm* the relative conductivity is 0.25. The relative conductivity increases to 0.31, 0.36, and 0.47 for film thicknesses of 4, 6, and 11 *nm* (see $\ast$ in Fig. 4). The reduction in conductivity, when compared with the bare Cu film value, can be understood from the electronic structure of isolated HGr. An ordered configuration of adsorbed hydrogens induce magnetic moment on Gr lattice by generating a localised state in the vicinity^35^. The generated magnetic moment is a result of an unpaired electron on the non-hydrogenated C atom. As mentioned in the previous section, the equilibrium distance between HGr and Cu film is nearly 2.30 Å. Such a small distance leads to strong interaction between the C[*p*_*z*_] and Cu[*s*] orbitals near E_*f*_ resulting in spin unpolarised HGr on Cu film. Thus, the contribution of *s* orbitals to the surface states decreases which leads to decrease in electronic conductivity of Cu films upon HGr encapsulation. In the following text we analyze the effect of strong hybridisation on the charge transfer between Cu and HGr. So far, our DFT calculations show that pristine Gr is an excellent choice to encapsulate the Cu interconnects.

Interestingly, the relative conductivity curve for Co encapsulation follows the HGr line as shown in Fig. 4. The effect of Co encapsulation is shown by large filled circles. It is clear that the Co encapsulation also results in significant reduction in the electronic conductivity. As Co is a magnetic impurity in the Cu surface, it acts as a scattering centre for the conduction electrons at the Cu-Co interface. The electronic conductivity of Co encapsulated Cu film of thickness *t* = 2 *nm* decreases by 38% when compared with the bare Cu film value. The reduction in conductivity value decreases when the film thickness increases from 4 *nm* to 11 *nm*. Our calculations show that Co encapsulation decreases the conductivity by atleast 15% for 11 *nm* thick Cu film. It is speculated that a thin cap of cobalt would be better for advanced Cu interconnects. Due to its stronger interaction with surface Cu atoms, Co adversely affects the electronic structure of Cu films. Similarly, Cu films when encapsulated with an atomic layer of Ru show reduction in the relative conductivity. The conductivity of Ru encapsulated Cu lies in between Co and Gr encapsulations. A recent study by Huang *et al*.^47^ showed that the Cu resistivity is higher with Ru liner than Co liner. The higher resistivity was attributed to interface scattering and possibly grain boundary scattering. In the present work, the possibility of grain boundary scattering is negligible as the thin films are atomically smooth and ideal. Additionally, in our simulations we do not consider effect of inelastic scattering at the interface. In sharp contrast, an atomic layer of Mo or Ta shows favorable effects on conductivity as compared to Co and Ru. For example, Mo encapsulation increases the relative conductivity of 2, 4, 6, and 11 *nm* thick Cu films by 21, 9, 5, and 2%, respectively. The Ta encapsulation also shows maximum increase in the relative conductivity for 2 *nm* thick Cu film. The increase is close to 14%. For larger thicknesses 4, 6, and 11 *nm*, the increase in the relative conductivity is 5, 3, and 1%, respectively. We see that the effect of Mo and Ta is very similar for larger thicknesses. Thus, an atomically thin layers of transition metals, Mo and Ta, show favorable effects on the electronic conductivity of Cu films.

**Figure 5:**
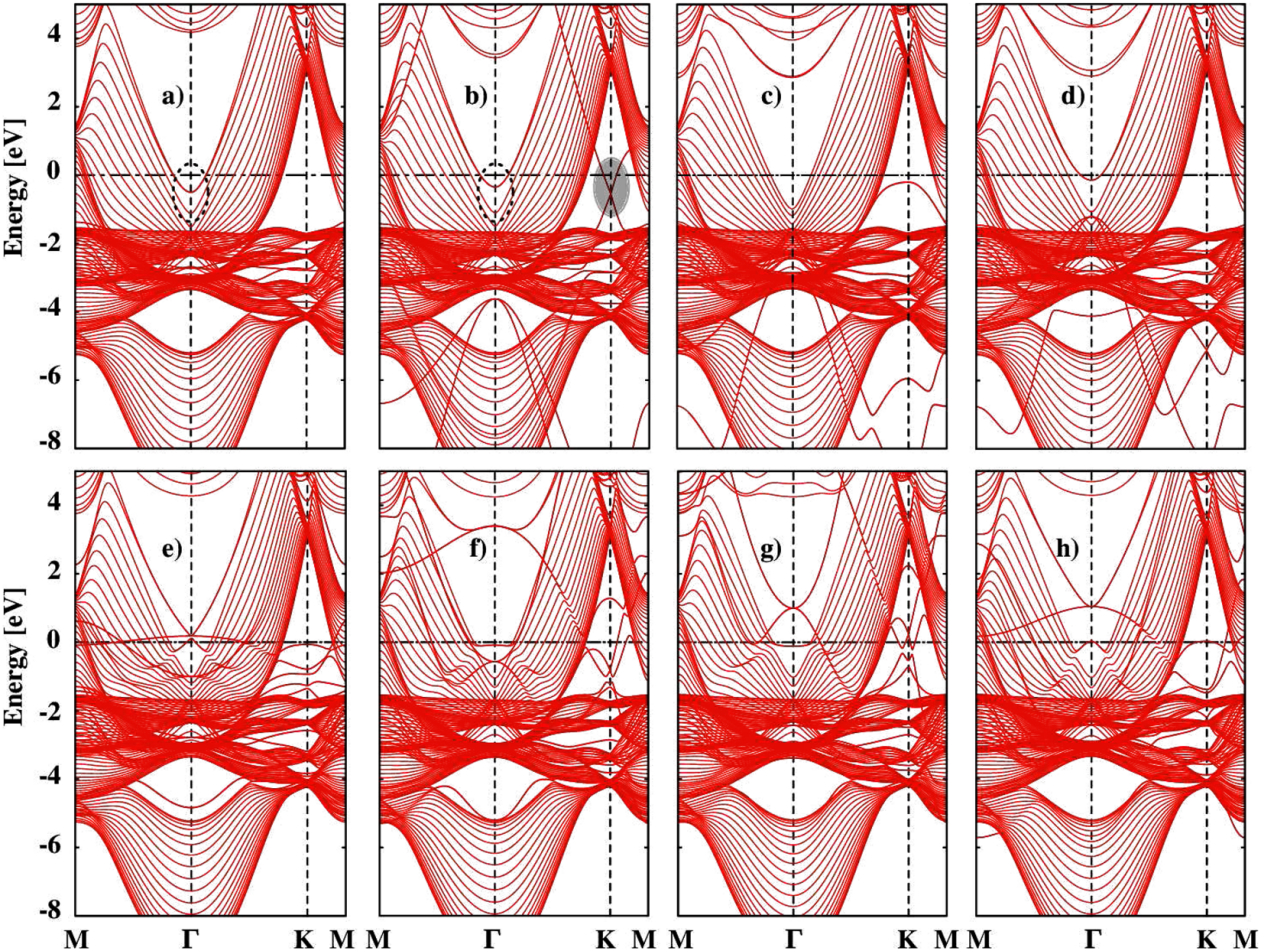
Electronic band structure along the high-symmetry lines in the Brillouin zone for *t* = 4 *nm* Cu film (**a**) bare (**b**) Gr (**c**) HGr (**d**) Graphane (**e**) Co (**f**) Mo (**g**) Ta and (**h**) Ru encapsulations. The horizontal dashed line indicates Fermi energy (=0 eV).

In order to understand the changes in the conductivity of Cu films upon encapsulations with 2D materials and transition metals, we investigate the electronic band structures. In particular, we consider the simple Drude model for electronic conductivity, *σ* = *ne*^2^*τ*/m* where *n*, *e*, m* and *τ* are carrier density, electronic charge, effective mass, and the relaxation time, respectively. By using this equation, one can understand the effect of m* and *n* on the conductivity of Cu films. The calculated effective mass m* for different encapsulations is reported in Table 1. The electronic band structure for a selected bare Cu film with thickness *t* = 4 *nm* as shown in Fig. 5a reveals delocalised occupied states at −0.48 eV at the high-symmetry Γ-point. The parabolic nature of the bands, shown in dotted elipse, results in 0.35 *m*_*e*_ effective mass for electron states at Γ-point. Our calculated value for the effective mass matches well with the theoretical value, 0.38 *m*_*e*_, reported by Cuong *et al*.^29^ and González-Herrero *et al*.^21^ for Cu(111) surfaces. The scanning tunneling microscopy (STM) measurements^35^ performed at 5 K revealed 0.39 ± 0.01 effective mass for the occupied electron states near E_*f*_. By utilizing a high-resolution angle-resolved photoelectron spectroscopy, Reinert *et al*.^48,49^ reported an effective mass m* = 0.41 *m*_*e*_ for the (111) surface of Cu. Upon Gr encapsulation, the electronic structure of both Cu film and Gr are well preserved as shown in Fig. 5b. One can easily identify the Dirac cone in graphene (see the shaded elipse). However, the Dirac cone is moved by ~0.50 eV below E_*f*_. This is due to the charge transfer from Cu film to graphene. As a result, the electronic states near E_*f*_ in Cu are shifted upward by +0.15 eV (see dotted elipse). A recent scanning tunneling spectroscopy (STS) data^21^ reported an upward shift of +0.13 eV on the onset of Cu(111). The Gr encapsulation increases the effective mass m* from 0.35 *m*_*e*_ to 0.41 *m*_*e*_ at Γ-point. Similar small increase in effective mass is reported^35^ in the STM study. This is due to the weak interaction between Gr and Cu film. The strong interaction between HGr and Cu film modified the electronic structure of both Gr and Cu film (see Fig. 5c). The Dirac cone in Gr is completely destroyed. We see a partial flat band at K-point near E_*f*_ which is due to localised states in HGr. The occupied states at −0.48 eV in bare Cu film (Fig. 5a) are moved above E_*f*_ due to charge transfer from Cu film to HGr. However, the effective mass calculated for the parabolic band below E_*f*_ at the Γ-point is decreased to 0.17 *m*_*e*_. The decrease in m* is due to the large curvature of the subband at energy −0.97 eV. In contrast, Graphane encapsulation resulted in increase in effective mass to 0.58 *m*_*e*_ due to small curvature of the subband (see Fig. 5d). Due to small change in the effective mass upon weakly bonded 2D materials, the change in relative conductivity of Cu film is very small.

**Table 1:** Calculated effective mass for the parabolic band below E_*f*_ at the Γ-point for different encapsulation materials (EM).

EM →	Bare Cu	Gr	HGr	Graphane	Co	Mo	Ta	Ru
m*/*m*_*e*_	0.35 (0.38^21,29^)	0.41 (0.41^21^)	0.17	0.58	3.29	0.56	0.16	0.16

The curvature of occupied bands near E_*f*_ decreases further when encapsulated with Co. For Co encapsulation, we find effective mass m* = 3.29 *m*_*e*_ which is much larger than 0.35 *m*_*e*_ in bare Cu film. Obviously, the electronic conductivity of the Cu films decreases. In addition, the electronic band structure reveals quantum oscillations at Γ-point (see Fig. 5e). Upon Mo encapsulation, the effective mass for the parabolic band at Γ-point is 0.56 *m*_*e*_ which is much smaller than in Co encapsulation. Therefore, Mo encapsulation shows favorable effects on the conductivity of Cu films as *σ* is inversely proportional to m*. The dispersion of the subband near E_*f*_ looks very similar when encapsulated with Ta and Ru. This leads to similar effective masses of 0.16 *m*_*e*_. However, their effect on the conductivity is very different which have addressed in the following text.

**Figure 6:**
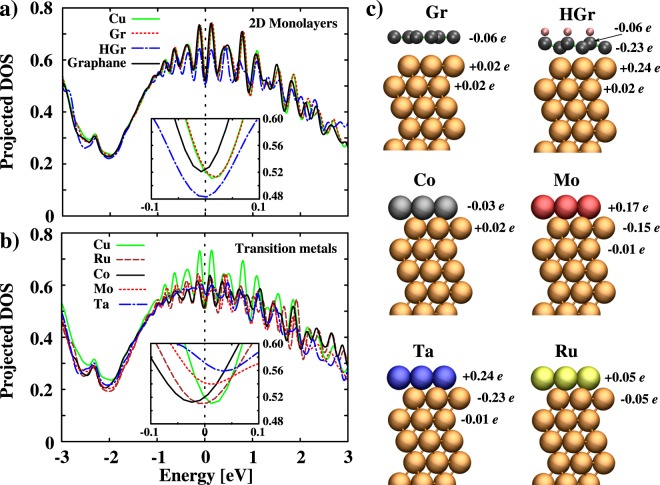
Projected density of states for Cu thin film of thickness *t* = 4 *nm* when encapsulated with (**a**) 2D materials and (**b**) transition metals (Gaussian broadening = 0.10 eV). The contribution near E_*f*_ (=0 eV) is shown in the insets. (**c**) Bader analysis of Cu thin film when encapsulated with Gr, HGr, Co, Mo, Ta, and Ru to show charge transfer between encapsulation and surface Cu atoms. The excess charge is indicated by negative (−) sign and charge depletion is indicated by positive (+) sign.

To obtain deeper insight into the electronic structure, specifically how the contributions of surface atoms depend on the encapsulating material, we analyze electron DOS for the copper film with thickness, *t* = 4 *nm* upon encapsulations. One can assume the existence of bulk-like charge distribution in the middle of Cu film with *t* = 4 *nm*. However, the effect of different encapsulations can be very different including a change in the Fermi surface, change in electronic DOS at *E*_*f*_, and change in surface potential. To better understand the effect of encapsulations, we focus on the changes in the electronic states around *E*_*f*_. In Fig. 6, we have plotted the projected DOS (PDOS) decomposed into *s* orbital contribution of Cu atoms. One may argue that the localised *d*-states in Cu do not contribute to the transport properties. Therefore, we do not take into account the contribution of *d*-states. Near *E*_*f*_, the *s*-states are delocalised and act as free-electron-like states. It is well known that the Shockley surface states in bare Cu films show major contribution around *E*_*f*_ (see green curve in Fig. 6a) and dominate the transport properties of the films significantly. Upon Gr encapsulation, the states near *E*_*f*_ are well preserved (see black curve in Fig. 6a). Due to the weak interaction between Gr and Cu film, the modulations expected in the PDOS near *E*_*f*_ are negligible. Moreover, due to the difference in their work functions, a little charge is transferred from Cu to Gr (see Fig. 6c). In contrast, due to strong bonding between Co and surface Cu atoms, the PDOS near *E*_*f*_ is affected significantly. The decrease in DOS at *E*_*f*_ leads to decrease in electronic conductivity as *σ* is directly proportional to *n*. However, one may argue that only decrease in *n* cannot affect *σ* significantly. Indeed, our calculated electron effective mass in Co encapsulated Cu film is much larger than in bare Cu film. Moreover, the relaxation time (*τ*) in thin metallic films is also a function of film thickness^50^. For thinner films, increase in successive collisions lead to decrease in relaxation time. Similarly, with Ru encapsulation the PDOS near *E*_*f*_ is reduced. This leads to the possibility of decreased conductivity. A recent computational study by Zahid *et al*.^14^ showed that the resistivity of thin Cu films coated with Ru barriers increases. The increase in resistivity was attributed to increase in diffuse scattering at Cu-Ru metal interface. In sharp contrast to Gr, the reduced PDOS near *E*_*f*_ for Cu films when encapsulated with HGr resulted in decrease in effective electronic conductivity. We can see a reduction in DOS for the Cu film upon HGr encapsulation. The reduction is due to a significant charge transfer from the Cu film to HGr (see Fig. 6c). The encapsulations with transition metals, Mo and Ta, show a charge transfer to the Cu film. As increase in DOS near *E*_*f*_ increase the electronic conductivity, Mo and Ta show favorable effect on conductivity. Therefore, we see an increase in PDOS at *E*_*f*_ for Mo and Ta (see Fig. 6b). For Ru encapsulation, the PDOS is slightly decreased when compared with bare Cu film. From the electronic structure analyses, we conclude that an atomically thin layer of Mo and Ta show more favorable effect on conductivity when compared to Co and Ru.

## Conclusion

We investigated, within DFT and semi-classical Boltzmann transport theory, the effect of graphene, partially hydrogenated graphene, graphane, Co, Mo, Ta, and Ru encapsulations on the electronic transport properties of Cu(111) thin films of thicknesses up to 11 *nm*. An atomically thin encapsulation of Co increases the film resistivity since Co atoms serve as scattering centres. We found that Co encapsulation decreases the effective electronic conductivity by at least 15% for 11 *nm* thick film. The decrease in conductivity is attributed to an increase in electron effective mass. On the other hand, Mo, Ta, and Ru have more favorable effect on conductivity when compared to Co. Atomically thin 2D materials, namely conducting graphene and insulating graphane both retain the conductivity of Cu films whereas partially hydrogenated graphene results in reduction of surface DOS and a reduction in Cu film conductivity. Our calculations suggest that pristine graphene sheet is an excellent choice as protective coatings for advanced Cu interconnects. These findings are of great interest to the materials and device community as it provides fundamental understanding of how encapsulation layer influences the electronic properties of thin Cu films.

## Methods

All calculations are carried out using the Vienna *Ab initio* Simulation Package (VASP)^19^ to perform periodic density functional theory calculations. The electron-ion interaction is treated within the projector-augmented-wave pseudopotential-plane-wave method^51^ and the Perdew-Burke-Ernzerhof functional^52^ is used for the exchange-correlation energy. The weak vdW dispersion interactions are taken into account by involving the DFT-D3 method by Grimme *et al*.^53^. The kinetic energy cutoff for the valence wave functions is taken to be 400 eV. The atomic positions are optimised until the forces on each ion becomes less than 0.001 eV/Å. A vacuum region of ~20 Å is used to avoid interaction between surfaces in the *z*-direction. We used a Monkhorst-Pack^54^ *k*-point grid of 24 × 24 × 1 to sample the Brillouin zone. The effective mass at the occupied parabolic band near E_*f*_ was calculated using the equation

1 $$\frac{1}{{m}^{\ast }}=\frac{{d}^{2}E}{d{k}^{2}}$$

An insight into the transport property, electronic conductivity, is obtained by exploiting the semi-classical Boltzmann transport equation as implemented in the Boltztrap code^39^. The code uses only the band and *k*-dependent quasi-particle energies, as well as intra-band optical matrix elements and scattering rates, as input. The relaxation time, *τ* is dependent on both the band index and the *k* vector direction. The output of the code, transport tensors, are linked to the fundamental electronic structure of a material. For electronic conductivity calculations within the Boltztrap code^39^, the *k*-grid was increased to 60 × 60 × 1. Such a fine grid yields accurate results. The electronic conductivity is reported for room temperature.

## Supplementary information

Supplementary Information
