# Supplementary material for: Effect of encapsulation on electronic transport properties of nanoscale Cu(111) films
Source: Sci Rep. 2019 Mar 5;9:3488. doi: 10.1038/s41598-019-40193-6 (PMC6401372; doi:10.1038/s41598-019-40193-6)
Supplement: Supplementary file 1 — Supplementary Information [file 41598_2019_40193_MOESM1_ESM.pdf]

Effect of encapsulation on electronic transport properties of nanoscale Cu(111) films

Prashant P. Shinde[1,*], Shashishekar P. Adiga[1,*], Shanthi Pandian[1] , K. Subramanya Mayya[1] , Hyeon–Jin Shin[2] , and Seongjun Park[2]

[1]Materials Simulation (SAIT-India), Samsung R&D Institute India – Bangalore
[2]Inorganic Material Lab, Samsung Advanced Institute of Technology, Suwon 433-803, Republic of Korea
[*]prash.shinde@samsung.com, shashi.adiga@samsung.com

Table S1. Calculated change in the *z* coordinate of Cu atomic layers in 4 *nm* (20 atomic layers) thick film upon different encapsulations. Δ_{zi}, indicates the change in Å for the ith layer calculated *w.r.t.* its bulk position. Only changes on one side from the centre of the Cu film are indicated. The two surface layers correspond to layer number 1 and 20. The negative sign indicates the inward relaxation of an atomic layer in the Cu film.

Layer No.	Bare Cu	Gr	HGr	Co	Ru	Mo	Ta
Δ_{20}	-0.40	-0.41	-0.37	-0.40	-0.38	-0.37	-0.40
Δ_{19}	-0.37	-0.37	-0.35	-0.37	-0.34	-0.34	-0.36
Δ_{18}	-0.33	-0.33	-0.31	-0.33	-0.31	-0.30	-0.32
Δ_{17}	-0.28	-0.28	-0.27	-0.29	-0.26	-0.26	-0.28
Δ_{16}	-0.24	-0.24	-0.22	-0.24	-0.22	-0.22	-0.23
Δ_{15}	-0.20	-0.20	-0.19	-0.20	-0.18	-0.18	-0.20
Δ_{14}	-0.15	-0.15	-0.14	-0.16	-0.14	-0.14	-0.15
Δ_{13}	-0.11	-0.11	-0.10	-0.11	-0.10	-0.10	-0.11
Δ_{12}	-0.07	-0.07	-0.07	-0.07	-0.06	-0.06	-0.07
Δ_{11}	-0.02	-0.02	-0.02	-0.02	-0.02	-0.02	-0.02
